# Motivations and Barriers for Veterinarians When Facilitating Fertility Management on UK Dairy Farms

**DOI:** 10.3389/fvets.2021.709336

**Published:** 2021-09-29

**Authors:** James John Brocket, Emma Fishbourne, Robert Frank Smith, Helen Mary Higgins

**Affiliations:** ^1^Craig Robinson Veterinary Practice Ltd., Carlisle, Cumbria, United Kingdom; ^2^Department of Livestock and One Health, Institute of Infection, Veterinary and Ecological Sciences, University of Liverpool, Liverpool, United Kingdom

**Keywords:** engaging farmers, proactive, preventative, behaviour change, cattle, fertility, health plan, inter-personal relationships

## Abstract

It is economically essential, but challenging, for dairy farmers to manage bovine fertility. Vets can help farmers to improve fertility, and this is cost-effective bringing benefits for production, animal health and welfare, and the environment. However, the extent to which vets are involved in fertility varies considerably between farms, for reasons that are unclear. This study investigated the motivators and barriers that vets perceive when trying to increase their involvement with fertility management on UK dairy farms. Interviews were conducted with 20 vets and four themes identified. The first, “clinical baggage,” highlighted vets' disillusionment due to past experiences of low uptake of their advice by farmers. Consequently, some vets made assumptions about farmer needs and behaviours, and exhibited ageist stereotyping. These issues, along with concerns and fatigue associated with repeatedly offering the same advice which was not acted upon, negatively influenced vets' engagement with farmers. The second theme “stuck in the comfort zone” revealed a loss of enthusiasm by some senior vets, whilst others lacked confidence to engage due to perceived gaps in their knowledge. Vets also reported farmers not perceiving their problems and lack of farm data or facilities, as barriers. The “vet-farmer relationship” theme highlighted building trust and developing strong relationships which were key drivers for vets to proactively engage and to “go the extra mile” for their clients. The final theme “money matters” explored vets' motivations to improve their clients' profitability and included the future sustainability of their own businesses. Our themes provide useful insight into the challenges vets face and provide key areas that can be targeted in future interventions to improve veterinary involvement in fertility management. For example, post-graduate training and support for vets needs to consider factors such as reflection, mentorship, stereotyping, relationships, communication, and leadership skills. This type of postgraduate support is currently limited for vets and requires investment from stakeholders if improvements in production, animal health and welfare, and the environment are to be achieved. Our findings are informative for facilitating veterinary involvement in any disease context, and are relevant for stakeholders including governments, educators, charities, farmer representatives, environmentalists, and veterinary leaders.

## Introduction

Reproductive efficiency is a key requirement that underpins the profitability and sustainability of any UK dairy farm. It has been well-established that sub-optimal fertility will return less revenue for the farmer through loss of production and higher replacement costs due to involuntary culls from barren cows (1). The underlying causes of poor fertility are multifactorial and vary between farms. However, they often involve sub-optimal nutrition, husbandry or breeding practises, as well as common endemic diseases such as lameness. Therefore, if farmers can successfully manage their herd's fertility and take preventative measures to pre-empt and mitigate the underlying problems, then in addition to financial and production gains, there will be animal health and welfare benefits as well as improvements in terms of reducing the environmental impact of farming.

Unfortunately, however, successfully managing fertility is challenging in practise for dairy farmers, and over recent decades the reproductive performance of the UK national herd has significantly declined (2, 3), although in the last 11 years there is some evidence that this may be stabilising (4). It has been shown that involvement with a veterinarian (vet) can help farmers to improve reproductive performance and that this is cost-effective for the farmer (5, 6). In practice, however, veterinary involvement in fertility work on dairy farms is highly variable, with different levels of engagement seen. At the lowest level of engagement, there are some farmers who do not involve their vet at all, followed by others who will involve the vet on an *ad-hoc* basis, usually in response to flare up of a specific problem (e.g., abortion outbreak). A step-up from this, are those farmers who have so-called “routine fertility visits” (RFVs) whereby the vet attends the farm on regular basis specifically to address fertility related issues.

However, even for farmers with RFVs, there is still significant variability in how involved their vet is, and how proactively fertility (and its underlying causes) are being managed. For example, some farmers with RFVs will be using their vet predominantly (or exclusively) for pregnancy diagnosis or for targeted treatment of sub-fertile cows, with the vet having minimal or no input into other aspects of fertility related issues. At the other end of the scale, some farmers with RVFs will be using these visits to engage fully with their vet in fertility “herd health management programmes,” also known as fertility “herd health planning” (FHHP). FHHP refers to the highest level of fertility management, and involves taking a proactive, holistic, and preventative approach. Hence, it requires close and on-going monitoring of farm performance data and management approaches, undertaking activities that seek to pre-empt or alleviate underlying causes of poor fertility, and regular reviews of progress with actions amended accordingly. FHHPs are highly farm specific and individually tailored because each farm has its own unique husbandry system, breed(s) of cow, and combination of risk factors (e.g., diseases, genetics, nutrition etc) that all impact upon fertility. This in turn means that the time, energy and money required to initiate and sustain a FHHP by the farmer and vet, will vary considerably depending on the individual farm, the specific challenges it faces, and how the farm's veterinary practice charges for providing these services. Readers who are not familiar with the details of FHHPs can obtain further information from references such as Hudson et al. (1), Smith et al. (7), and Smith (8). Executing a FHHP does not necessitate veterinary involvement *per se*, however, due to the clinical and often complex, multifactorial nature of the work, vets are ideally placed to help farmers initiate and implement FHHPs in practice.

There is a lack of specific data regarding the proportion of UK dairy farmers who currently use their vet for fertility in the different ways just described. However, in a qualitative study, vets reported that <10% of their clients were engaged in an effective preventative disease programme (9). Although this was not specifically fertility related, it provides some insight into the involvement of veterinary surgeons in an advisory role. In addition, but again not specific to fertility, research has suggested that only ~48, 40, and 31% of UK farm vets were involved in herd health planning for mastitis, Johne's disease and lameness, respectfully, at the most involved level possible (10). Overall, and given that the fertility of the UK's national herd has declined in recent times, it seems reasonable to propose that there is more scope for veterinary involvement in the fertility management of dairy herds, whether that is commencing RFVs on farms that currently do not have one, or increasing the level of involvement on farms that already have RFVs to implement FHHPs in full. This view is also corroborated by the combined clinical experiences and professional networks of the authors.

Looking from the farmers perspective, there has been some research into the motivators and barriers for farmers implementing various veterinary led, but not fertility specific, initiatives (11–15). A further study that surveyed all year round dairy farmers specifically in the context of fertility, revealed that farmers rated proactivity as a key attribute they look for in a vet (16). Another study by Svensson et al. (17) highlighted that trust, feasibility and priorities were the main factors for adherence and non-adherence by farmers, for veterinary led initiatives. The information from these studies gives important insight from the farmers perspective with respect to their engagement with veterinary led preventative herd health programmes.

From a veterinary perspective, there are several advantages for vets if they can engage with all their clients to successfully deliver FHHPs. This includes an increased probability of having more satisfied clients and healthy herds, which in turn is likely to improve the vet's own job satisfaction. The ability to effectively deliver FHHPs is also likely to make veterinary businesses more sustainable because they will have another revenue stream in addition to emergency work and medicine sales. In addition, they are more likely to have clients long-term because farm businesses will be more profitable and farmers may be less likely to exit the industry. Thus, there appears to be significant motivation for vets to engage with farmers. It is interesting, therefore, because despite some seemingly major advantages for vets, the reasons why vets do not have greater involvement in fertility and in particular in delivering FHHPs, remains unclear.

Overall, therefore, while there has been research exploring the farmer's perspective on the uptake of various veterinary led initiatives, there is a lack of research into the barriers and drivers that vets perceive and experience with respect to increasing their involvement in fertility on their clients' dairy farms. This study aimed to identify these motivations and barriers, with a view to enabling steps to be taken to facilitate improved veterinary engagement with dairy farmers.

## Materials and Methods

### Selection Criteria

Vets were selected based on the following criteria. They had to be practising in the UK with an average daily clinical workload consisting of at least 30% dairy cattle work. In addition, participants had to be at least 2 years qualified. Veterinary practises were pragmatically selected based on location in the north west of England or south west of Scotland. Practises with eligible vets were identified based on the knowledge and networks of the research team and by utilising the Royal College of Veterinary Surgeons (RCVS) online search facility (https://findavet.rcvs.org.uk/home/). Potential practices were contacted by telephone, the project was explained and general permission sought from the managing directors for participation of their vets. Individual vets were then purposively selected to give a broad range of demographic characteristics, specifically, to give a balanced range of clinical experience, role within the practice and gender. A sample size of ~20 vets was planned with interviews undertaken until data saturation occurred (18, 19), and following discussion amongst the research team. The research team agreed saturation had occurred by the 20th interview. Ethical approval for the study was granted by the University of Liverpool Veterinary Ethics Committee (reference number VREC791). This study has been reported in keeping with the COREQ checklist i.e., the consolidated criteria for reporting qualitative studies (20).

### Data Collection

Before the interviews took place, each vet was emailed and read the participant information sheet, which included details about the background and purpose of the research, as well as about the interviewer (JB) who was an experienced mixed practice vet, studying for a post-graduate qualification. They also signed the consent form and completed a short questionnaire to capture demographic information (see Supplementary Material for details). All interviews were undertaken by JB, face to face, between June to December 2019, at the place of work of the vets. All interviews were audio recorded in a private room with no one other than the interviewer and participant present.

Interviews were semi-structured. This allowed a comparable framework yet facilitated deeper discussion and exploration of ideas through reactive questioning to points made by participants. The interview structure was constructed using critical incident methodology (21). The use of this technique prompted participants to discuss situations that they had been personally involved in which aids thinking and the generation of a rich dataset. Specifically, participants were asked to consider two very different dairy clients, one for whom they were currently delivering their RVF, and another client that they were either never, or rarely, involved in any aspect of their fertility management. Participants were asked to describe what actions, if any, they had taken to further increase their involvement with each of these two clients in fertility work, and to reflect on their experiences, including why they had not attempted to engage with them further, if this was the case. They were also asked to describe how these experiences reflected their clients in general, and how their experiences shaped the way they offered services to their clients. Other topics discussed included their relationship with their clients and the education they had received on fertility. The majority of questions were open to encourage discussion on each topic. The full interview schedule is provided in the Supplementary Material.

Pilot interviews were undertaken first on 2 vets. This data was not included in the final analysis but it allowed the questions to be refined and provided an estimate for the duration of the interviews. No incentives were offered to the participants.

### Data Analysis

Interviews were transcribed verbatim by a third party and then cross checked by JB; they were not returned to participants and participants did not provide feedback on the results. Qualitative analysis was performed on the dataset using NVivo software (version 12, QSR International Pty Ltd). The analysis used an inductive thematic approach for exploratory analysis (22), with themes derived from the data. Using the software, the text in the data was coded initially by JB. Codes included sentences or part of, or whole paragraphs that corresponded to a succinct idea or belief. After all transcripts were coded, codes were merged based upon their similarity. This merging process brought together themes which covered all the transcripts and summarised the key concepts. Transcripts were re-read to confirm that the themes accurately represented the dataset. The themes encapsulated the dataset and allowed conclusions to be made and the aim of the study to be explored. JB led the analysis, with coding and themes continuously developed and refined through discussion with HMH and EF.

## Results

### Characteristics of Participants

In total, 19 veterinary practices were contacted to participate, all of which agreed to take part. Due to time constraints, however, 15 practices were used for interviews. Of the 21 individual vets who were asked to participate that fitted the required selection criteria, 20 agreed to participate. There were 13 men and 7 women. Ages of participants ranged from 27 to 55 years old with a mean of 37 years. Mean number of years qualified was 13 (range 3–31). There were 8 assistants and 12 senior partners/directors. Of the 20 participants, 10 were working in mixed practice and 10 were in farm-only practice, and the mean percentage of clinical time that participants spent working with dairy cattle was 62%, ranging from 30 to 98%. In total, 4 had post-graduate cattle-related qualifications. The size of the veterinary practice they worked in varied from 3 to 28 farm vets, with a mean of 9. Interviews lasted between 25 and 44 min. The number of dairy farms for which participants were currently personally involved in delivering fertility management programmes ranged from 2 to 20 (mean of 8 farms).

### Themes

**Figure 1:**
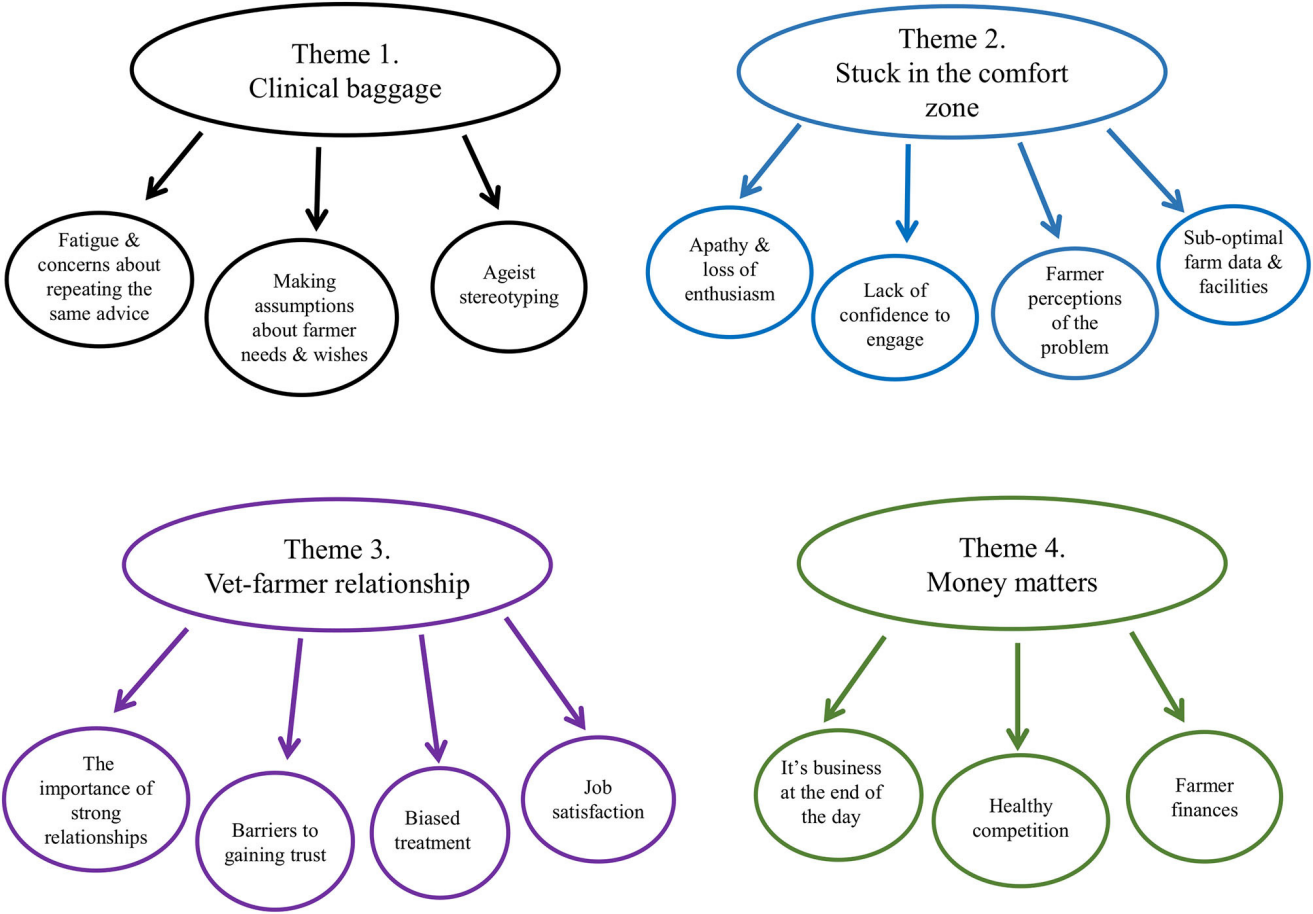
Schematic overview of themes and sub-themes generated.

The analysis generated 4 main themes namely “Clinical baggage,” “Stuck in the comfort zone,” “Vet-farmer relationship,” and “Money matters.” Each main theme contained sub-themes, as illustrated in Figure 1. Each theme is presented in turn, using exemplar quotations from participants. To maintain anonymity, names of participants have been replaced with pseudonyms.

#### Theme 1. “Clinical Baggage”

A strong theme expressed by the participants was how past experiences shaped and sometimes seriously hindered their willingness to engage with farmers. In other words, clinical experience could act as “baggage.” There were three sub-themes each of which are explored in turn: “Fatigue and concerns about repeating the same advice,” “Making assumptions about farmer needs and wishes,” and “Ageist stereotyping.”

##### Sub-theme 1.1 Fatigue and Concerns About Repeating the Same Advice

Some of the participants felt initially enthused and motivated to engage with dairy farmers regarding their fertility. However, often when advice they gave was not acted upon by the farmer, they felt awkward and reluctant to offer the advice repeatedly. For example:

*You mentioned it the previous week. You mentioned it the week before. So, you do sort of lose the will to keep saying the same things over and over again. (Gillian, 30, assistant)*

An element of this awkwardness and reluctance to repeat recommendations was due to how the vet feared they may be perceived by the farmer. Specifically, some participants were concerned that they may be perceived by the farmer to be overly promoting their services for the benefit of themselves or their practice, and not for the client. As Ashley described it:

*You'd never want to be seen as a salesperson. (Ashley, 28, assistant)*

##### Sub-theme 1.2. Making Assumptions About Farmer Needs and Wishes

Due to previous negative experiences with poor uptake of advice, some interviewees were reluctant to engage with other clients on the subject of fertility. The concern for some participants is that they believe new farmers are likely to respond in a similar way to those that rebuked offers of assistance from the vet in the past. Some vets made assumptions about what they thought farmers knew and wanted, such as Josh:

*We assume that every client knows that*. [what fertility services the practise offers] *We do lots of routine fertility work and we assume the guys that are happy are happy, and the guys that are not interested are not interested. (Josh, 39, Director,)*

Moreover, David made it explicitly clear that if, historically, little preventative medicine work has been carried out for a client and they have not previously shown interest in this area, then he personally would be less likely to proactively offer fertility services to them, as he commented:

*I've probably not engaged as well as I might have done, thinking about it, but that's probably because the farmer is very much- Yes, probably they haven't engaged so I haven't, so yes. (David 29, assistant)*

##### Sub-theme 1.3. Ageist Stereotyping

Based on previous experiences, some of the participants also highlighted that they thought that the older generation of farmers would be unlikely to engage or to be interested in the fertility services that the vet could offer them, even though it could improve their profitability. As such they often did not broach the subject as they believed nothing would come of it, as Marvin explained:

*A lot of the older generation of farmers are happy just to have the farming way of life how it is, and some years you make money and some years you don't. (Marvin, 49, director)*

Along these lines, some interviewees felt more encouraged and motivated with younger farmers as they perceived that a younger farmer would be interested and more receptive to what the vet had to say. Dafyd highlighted this point as follows:

*It's fantastic to have a younger farmer that is really wanting to go ahead and actually make a profit out of the business and make a living out of the business, as opposed to the old duffer that is just winding his way down to retirement. (Dafyd, 50, director)*

Thus, vets sometimes used age of the farmer to explain why they thought farmers were engaging with them, or to explain why they were not, themselves, being more proactive in engaging with some farmers.

#### Theme 2. Stuck in the Comfort Zone

Another theme that developed through the interviews was that the participants were often content to continue working without changing their status quo. Numerous reasons arose from different participants for why that may be. These are encapsulated by four sub-themes which were: “Apathy and loss of enthusiasm,” “Lack of confidence to engage,” “Farmers perceptions of the problem,” and “Sub-optimal farm data and facilities.”

##### Sub-theme 2.1. Apathy and Loss of Enthusiasm

Some participants who had not engaged with certain farmers on the subject of fertility, reflected that it came down to apathy as to why they had not done so and some spoke frankly:

*I don't think there's a direct answer as to why I've not. Probably laziness (David, 29, assistant)*

A number of experienced participants felt that their drive to engage and enact change with their clients had decreased as they got older. Many participants cited other commitments, demanding schedules, and that their priorities with respect to work had shifted elsewhere:

*I guess my challenge, as you get slightly older, 25 years into practise, is keeping that enthusiasm, that momentum…. sadly, if I am honest with myself, I don't think I am doing as good a job as I was doing 10 years' ago, because I am not as focused for a whole host of different reasons. Personal drive, other things that are coming onto your plate the whole time. (Marvin, 49, director)*

##### Sub-theme 2.2. Lack of Confidence to Engage

In contrast to the previous sub-theme, for some less experienced participants, whilst they expressed enthusiasm to discuss and engage with farmers about fertility, they had reservations about carrying it through. Although conflicted, they often ended up accepting the status quo without ever attempting to change it. This sometimes appeared to stem from a lack of confidence, often underpinned by a lack of self-belief in their own fertility knowledge, as Gabriel explained:

*If it doesn't work with that, then I feel like I'm going to plan B, which I don't know plan B as well, so I feel like I've probably got less conviction, less confidence in myself. (Gabriel, 28, assistant)*

In addition, the concern for a number of the vets interviewed was having information and evidence at hand to support themselves if they were challenged regarding what they recommended to farmers and hence, to be able to back up what they are saying. Having the ability to recall specifics was often desired but challenging, as Ashley said:

*Probably because I maybe haven't done enough research myself in that area. Like I read the odd things, but maybe I don't write it down or take it in well enough to then relay. Then because I'm not using it all the time, then it just goes out of my head. (Ashley, 28, assistant)*

The majority of participants said they did not feel prepared or in a position to provide in-depth fertility herd health management on farms when they left university. Many also felt that the mindset of their veterinary practise either helped develop or stifle their development, as summarised by Martin:

*My undergraduate teaching prepared me to be an ambulatory fire brigade service vet more than it did for fertility work and herd health work. I think even my first couple of jobs, that was very much the ethos….They were not progressive practices. I think new graduates would be very much benefited by being in a progressive practice. I think being in a practice that has a good ethos toward progressive dairy work, that's probably the thing that prepares you the most. (Martin, 33, assistant)*

The participants who had gained further post-graduate qualifications in cattle fertility, such as the Diploma of Reproduction (DBR), had garnered a lot of confidence from it. This gave them the conviction to approach and openly discuss with farmers the improvements that they could make:

*I think what the DBR did give me was the confidence. (Marvin, 49, Director)*

However, for one participant, even though he felt he now had the knowledge and hence confidence to effectively explain to clients the benefits of fertility programmes, this was still not always sufficient to overcome a fear of upsetting the farmer:

*It's the elephant in the room, that it's almost taboo to say, “Why don't you have a routine?” (Martin, 33 assistant)*

##### Sub-theme 2.3. Farmers Perceptions of the Problem

When vets did attempt to enter into discussions with the farmer over their herd fertility, they often reported what they felt were farmer perceptions of barriers, such as:

*That is a big problem. Farmers don't think they've got any time to do things. (Joanne, 37, director)*

Furthermore, one participant was aware that they were stuck in the comfort zone but were waiting until the farmer perceived there was a problem themselves, before engaging:

*I think it always comes to the fore when somebody has a bad fertility visit, that PD session, or a couple of bad PD sessions that they start looking for answers. There's always a prompt to engage, isn't there? (Josh, 39, director)*

On a similar note, some of the participants also felt that some farmers were not, or could not, perceive the problem at all, and this created challenges. As expressed by Alfie:

*If they're not admitting that they've got issues and recognising that, it's pretty difficult to get them on board with recommendations and things. (Alfie, 27, assistant)*

On the other hand, it could be the influence of the farmer that pushes the vet, as illustrated by this participant's comments:

*He's probably driving me rather than me driving him to a certain extent. That's good for me. It keeps me fresh to a certain extent. (Martin, 33, assistant)*

Interestingly, one participant, Hugh, felt that, fundamentally, it was not the vet's role to change the perceptions or outlook of farmers, especially those who are currently have poor fertility performance. Rather, Hugh felt that veterinary attention should instead be focused on farmers who are already more forward-thinking:

*But I don't think as vets we should waste our time trying to get those people to really bring themselves up. I think it's a wasted effort in general. (Hugh, 32, director)*

##### Sub-theme 2.4. Sub-optimal Farm Data and Facilities

It was also acknowledged that it can be difficult for vets to engage and explain to farmers where precisely fertility on their farm can get better, and the areas that the vet can help improve. In this context, participants sometimes struggled to get meaningful farm data. This was a reoccurring comment by many participants, as they felt they could not gain a foothold to highlight issues to the client, without having the farm records to back this up. This often came down to whether the farm milk recorded or not, as Alfie pointed out:

*It is more difficult in some farms that don't milk record, it's a bit more difficult to get involved with those ones. It is quite hard to crack, because you've not really got any data to go off. (Alfie, 27, assistant)*

In addition, farm facilities and perceived inconvenience in executing the tasks involved *per se*, were also noted as a barrier that vets felt existed, along with past experiences pass down from family members, as Gillian explained:

*It's faff for him. He doesn't want to do it. He wouldn't want to do it for all of his cows. He doesn't have the set-up to do it………Even trying to persuade him to do things like fixed-time AI is difficult because, “Oh, it never worked for my dad, so I don't do it.” (Gillian, 30, assistant)*

#### Theme 3. Vet-Farmer Relationship

This theme focused on inter-personal relationships, including their importance relative to veterinary knowledge and clinical skills, challenges developing relationships and gaining the trust of farmers, and the consequences of these relationships in terms of how intrinsically motivated vets were to engage with their clients and help them. There were 4 sub-themes: “The importance of strong relationships,” “Barriers to gaining trust,” “Biased treatment,” and “Job satisfaction.”

##### Sub-theme 3.1. The Importance of Strong Relationships

A strong theme throughout the interviews was about building a relationship with the farmers and it was acknowledged that clinical skills and technical knowledge alone were not sufficient:

*You need to be competent. But there are probably a lot of better vets out there that don't get on with any farmers because they haven't got the social skills. (Hugh, 32, director)*

Furthermore, developing this relationship was viewed as essential, as demonstrated by this participant, who frankly proclaimed:

*The most important thing is that if you are an arsehole, nobody wants you on their farm. You have to be likeable. (Peter, 29, assistant)*

Many of the participants were aware when they did not have a strong working relationship with certain farmers. However, this social awareness allowed them to avoid upsetting and straining the relationship further. Instead they would often delegate another vet to deal with fertility management on the farm, for example:

*There are farmers that I don't necessarily click with quite as well, and I'll send others to it because you know that they do and they'll get more done. They'll get results because the relationship is better already, just right from the word go. (Sandra, 40, director)*

Interestingly however, some of the interviews highlighted that even when the vet-farmer relationship was considered good, vets may not always tailor recommendations to suit individual farms, as Marvin commented:

*I mean everyone will be different, as you probably know. I think you've got to try and understand your client as to what makes them tick…. But sometimes I think we forget to ask our clients what they actually want. (Marvin, 49, director)*

##### Sub-theme 3.2. Barriers to Gaining Trust

It is evident from the previous sub-theme that developing a good working relationship with the farmer and being liked was seen as crucial. For the farmer to act on advice they were given, the special importance of gaining the farmer's trust was also specifically highlighted:

*I think the more they get to know you, the more they trust what you say, whereas if you just pitch up and they've never met you before, and you're saying they need to do this, this and this, then I think you're much more likely to encounter resistance to that. (Alfie, 27, Assistant)*

Furthermore, participants across all the demographics perceived that age and experience were definite barriers to gaining that trust. For example, experienced vet, Will, made this remark:

*Any young vets will tell you that they've had experience where they say something to a client, be it a pet client or a farm client, and it's not received or acted upon. Then somebody with a grey hair or a beard, or glasses, says the same thing and it is accepted. That's just a fact of life. (Will, 55, assistant)*

And this sentiment was echoed by a less experienced vet, David:

*But I do find that maybe my age, maybe the fact that I'm- I know it's specific to me, but maybe it's because I'm new and why would they trust me? (David, 29, Assistant)*

Many also believed that the trust would develop after a period time. For example:

*I guess on some farms it is still building up that relationship, that trust, and not necessarily with you, but just with the advice they're given really. Yes, it just takes time and it takes a relationship and if you don't have that, then that is where it makes it harder. (Nigel, 33, director)*

##### Sub-theme 3.3. Biased Treatment

A strong subject that shone through was that participants were far more motivated to engage and offer advice and instruction to clients that they both liked and got on well with, as Peter described it:

*You think you've got a good relationship, you almost, kind of, bend over backwards to help them out. (Peter, 29, assistant)*

Equally though, many felt reluctant to even try and engage with clients that they did not get on well with because of the personal cost to themselves with no perceived reward. As Gabriel explained:

*I probably work harder for the ones I like and get on with. I feel like there's a lot of time and effort you can put in, and if it's not appreciated then why bother? (Gabriel, 28, assistant)*

##### Sub-theme 3.4. Job Satisfaction

Many of the vets cited job satisfaction as a main motivator for them to engage with farmers to delivery fertility management programmes. This arose in many guises, but mainly centred around the relationship with the farmer and the fulfilment derived from helping their farmers improve the herd, or from making their clients lives easier, both professionally and financially. Gillian is an example of a participant that expressed this view, and she also highlighted the animal health and welfare benefits, which were important to her:

*So, it's probably just a case of you don't want to see them fail; you know, you don't want them to be losing money and getting into difficulties, and then the animals not get the care they need because they're strapped for cash, so they can't look after them, you know. (Gillian, 30, assistant)*

#### Theme 4. Money Matters

This theme focused on financial issues which permeated discussions throughout, and is reflected in 3 sub-themes, namely: “It's business at the end of the day,” “Healthy competition,” and “Farmer finances.”

##### Sub-theme 4.1. It's Business at the End of the Day

It was often noted that there was a financial incentive to engage with clients and build more advisory work around FHHPs, rather than rely on emergency work and medicine sales. This was exemplified by Josh, who reflected on changing the business model of veterinary practices to help bring in more revenue streams:

*We- as an industry, I think we're still incredibly reliant on drug sales, but, yes, we have to be valuing what we do, and we have to be looking at services and consultancy-based type services. (Josh, 39, director)*

Participants highlighted that FHHPs were an ideal opportunity to engage with farmers over other aspects of their farm and disease problems, which could help to increase the services offered and the finances of both parties:

*I try and do a whole farm approach so if there is – we pick up a massive lameness issue when you're doing a routine [FHHP], or you pick up a whites [endometritis]issue or whatever it is, or you think it's nutritional basis (Eliza, 45, director)*

Some participants were seriously worried about the long-term sustainability of their own businesses if they were unable to engage effectively with their dairy clients:

*If you don't adapt and look after your farm business… Lots and lots of mixed [veterinary] practices in the country, the farm side [of the veterinary business] has gone. You have to think about the financial aspects of the farm work because we're in a business. (Will, 55, assistant)*

This long-term sustainability was reiterated by several participants. Many were driven to engage more with clients not for short term financial gain but to support farms so there will be work for future years:

*If they [farmers] are more successful and they can be more efficient, probably efficiency is better than more successful, efficiency both financially and productivity wise, then they are going to be in that industry in 10 years' time, and I'm going to have an industry to work in. (Marvin, 49, director)*

Furthermore, finance was not just a consideration for the senior partners/directors who owned the business and would therefore see a positive return on their investment if things are going well. This was also a consideration for assistants, as Ashley explained:

*I really like the bosses and I suppose I really enjoy working here, so I want it to always be successful so there's always a job for me. (Ashley, 28, assistant)*

##### Sub-theme 4.2. Healthy Competition

As well as financial motivations *per se*, it was noted that competition from other vets encouraged participants to engage with clients. This was due to a fear of farmers becoming disillusioned with the service and changing to a rival practice. For example:

*Where I work, it is an exceptionally concentrated number of cattle practices around us. Some of them are quite vociferous, high-profile, ones. I personally have definitely always felt the heat of competition from them, which is probably a good thing. (Will, 55, assistant)*

However, some participants felt their businesses and services were more strongly rivalled by other farm advisors:

*I think other vets are- there's always competition with other vets. I think we worry far too much about other vets. And actually, the major competition is coming from the consultants, advisers, feed nutritionists. (Josh 39, director)*

##### Sub-theme 4.3. Farmer Finances

When money was raised or discussed, the participants spoke about the benefits for their own veterinary business if they could engage farmers in FHHPs. However, in addition to this, the overwhelming feeling was that their professional input would also be financially beneficial for the farmer, as Sandra highlighted:

*I think having you on the farm regularly for discussions of anything that recognise their fertility, is a massive value for them. (Sandra, 40, director)*

However, many such as Wallace felt this view of the cost-benefit of veterinary intervention, was not always shared by the farmer:

*He just didn't really see that he was losing any money from it [poor fertility] or not. He didn't see the gains that he could. He could see what the cost of a vet visit would be. (Wallace, 45, director)*

#### Other Barriers Reported by Participants

There were a few other barriers that participants reported, out with those encapsulated in the main themes. In particular, one barrier suggested by some participants was a lack of marketing to their clients on what their veterinary practice could offer regarding fertility services. In addition, it was acknowledged that a disjointed or disorganised approach to fertility by the practice hampered some vets' ability to engage. Lastly, some specific farm management practises, such as the use of bulls for natural insemination, made it more difficult for some participants to tailor their services specifically to the clients' needs.

## Discussion

This study focused specifically on the clinical context of increasing vets' involvement in fertility management on dairy farms, which varies considerably in practice. As previously mentioned, some farmers choose to have no veterinary involvement at all, whilst at the other end of the scale, some farmers engage as fully as possible with their vet to undertake fertility “herd health planning” (FHHP). FHHP can be considered a specific example of the broader concept of “veterinary herd health planning” (VHHP), which we take to mean any evidence-based, proactive, veterinary led management programme, that seeks to protect and improve animal health and welfare on farms, and in doing so, promotes farm productivity. VHHP achieves this by improving animal husbandry and/or preventing and controlling diseases. Throughout this discussion section, we have compared our findings elicited from vets in the context of fertility, to other social science research on non-fertility related veterinary involvement and the broader concept of VHHP, because we could not find any directly comparable studies.

Many participants felt disillusioned when their advice was not acted upon by the farmer and this has also been found in other studies (23). Our results support the view that it is important for vets (as both individuals, and as a profession) to fully understand why advice sometimes has no impact because this means not only that the advice is not acted upon in the short term, but it also serves to discourage vets from attempting to engage with farmers in the future. There are many possible reasons why advice may not be acted upon by farmers (24). This includes farmers not perceiving the problem in the same way as the vet, farmers not believing they have the ability, money, time or facilities to make the changes, being risk averse, or suffering themselves from human health problems that make implementation difficult. Farmers may also feel that the advice is not in keeping with their priorities or goals at the time (i.e., they may lack intrinsic motivation, peer pressure and social norms) or they may have previously tried but failed to implement the advice and now believe it to be impractical, not effective or even harmful. However, while many factors can be involved, we suggest that communication skills have an important role to play in this regard, and it has been highlighted that communication skills within the veterinary profession are sub-optimal at times (25, 26). In particular, there is evidence to suggest that sometimes vets only utilise one method of communication with farmers (25). In addition, there appears to be more scope for vets to adjust the way they interact and facilitate advice based upon each, individual, farmer. Tailoring how advice is communicated to individuals has been shown to be effective in behaviour change (27, 28). Furthermore, some participants felt that farmers themselves were the actual barrier to implementing their advice, and this may be due to competing priorities at that time for the client, as previously mentioned. Research has suggested that vets may be poor at ascertaining farmer goals (29). If vets are not listening or do not actively establish and review their client's current needs (which are likely to evolve over time) then this may make engagement more difficult both now and in the future.

In addition, participants sometimes made assumptions about farmers based on generalisations and this was often due to negative experiences with farmers and the disillusionment of poor uptake of their advice, historically. For example, sometimes this led to some vets in this study not offering advice to older farmers and instead targeting younger farmers. One study (*n* = 43 farmers) has supported the notion that younger farmers were more likely to have intent to implement a zoonotic control plan (30). However, this research did not establish a causal relationship and one plausible explanation for this finding is that vets are preferentially targeting younger farmers with their advice. A major disadvantage of tailoring advice to farmers based on their age is that some older farmers will be denied opportunities to improve the fertility of their herd, and it becomes a self-fulfilling prophecy, with implications for the equality of provision of veterinary health services. In our opinion, it is vitally important that ageist stereotyping does not bias vets when attempting to proactively engage with farmers.

Apart from age, vets in this study sometimes made generalisations based on how the farmer currently uses the veterinary practice for services. Specifically, if the practice is used primarily for emergency work, participants were quick to perceive that the farmer would not be interested in the practice's fertility services. However, this perception is shown to be contrary to previous research by Svensson et al. (31) which showed that herd health services were wanted by farmers, however they expected vets to initiate the conversation. Based on these findings, overall, it appears likely that there are opportunities for vets to engage further with farmers, if they proactively offer their services and advice to all clients regardless of prior experiences of working with them, patterns of service use or any other factors, and if not taken up, they attempt again via alternative methods.

It is also important, in our view, for vets to overcome any disillusionment they associate with the poor uptake of their advice, not least because it will help to counteract negative generalisations being made about different types of farmers. One way to help address this, is through self-reflection. Reflection has been shown to aid learning outcomes in the human medical field (32). Reflection during clinical case meetings within practices on both successful and unsuccessful engagement with farmers, could allow dissemination of gathered knowledge. This will enable the practice to formulate a concerted plan to aid future engagement, including helping to identify and mitigate discrimination based on age or other factors. It is important that these meetings are constructive and supportive. There is a risk that if there is no enthusiasm for change from senior vets and colleagues, then future engagement could be stifled and negative experiences with a farm may be reinforced. Reflection will become a compulsory component of continued professional development (CPD) from January 2022 by the Royal College of Veterinary Surgeons (RCVS).

Some participants perceived that their farmers had other priorities and this is echoed by another study investigating uptake of control plans by farmers for Johne's disease control (13). In this context, our findings also showed that effectively highlighting issues to farmers was an important hurdle to engaging farmers in FHHPs, and proved to be a significant barrier for vets to overcome in some instances. To facilitate highlighting fertility issues to farmers, one commonly used method is benchmarking the farm against the industry standards and drawing attention to areas that are under performing. However, benchmarking requires data. A quick, easy to access, source of fertility data for vets on an individual farm is through third party access to the farm's milk recording data, and there are several software programmes available to aid analysis of this information. Many participants reported a lack of milk recording on the farm as a barrier to engage about fertility. However, it is worth noting that some non-milk recording farms do have adequate or even excellent fertility records and data, but access and format mean it takes longer to extract the desired information from it. This study has also highlighted that the willingness to undertake this extra data mining is restricted by a lack of time that vets have to undertake it. This finding is supported with research that shows both vets and farmers lack time (33). While issues surrounding data and time pressures are barriers to engagement and FHHPs, they are not insurmountable. It has been proposed that the use of paraprofessionals and technicians would give a vet more time for advisory work (34).

Another strong theme that emerged was that participants often ended up in a status quo with their clients where they felt comfortable. Engaging with farmers on FHHPs sometimes forced vets to leave their comfort zone. This was an unexpected finding as fertility work on dairy farms has been a cornerstone of veterinary farm work for decades. Thus, even when farm data was available for the participants to use to help their clients, some were apprehensive to engage. Often a reason for this was their own lack of confidence, which was typically voiced by less experienced vets. This has been shown before, when sheep veterinarians believed they did not have sufficient knowledge (35). Adler et al. (36) demonstrated that higher degrees of technical knowledge by farmers were shown to favourably mediate behavioural change on farms. It is therefore important that the vet has strong technical knowledge in dairy cattle herd fertility in order to be in a position to pass that knowledge on to their farmers. It has been shown that vets who have undertaken post graduate qualifications and extensive continuous professional development (CPD) are far more likely to be involved in delivering herd health disease control programmes (10). It could be postulated from this study that a reason for greater engagement could be a higher confidence in the vet's knowledge on the subject. Some participants felt unprepared for progressive fertility management on dairy farms after they left University. This is likely to have led to lower confidence to engage with farmers. The emergence of more post graduate training and qualifications may help in this area but this will also be influenced by whether the vet wishes to pursue this area of work and is in a position to do so. This is highlighted in the disparity of hours of dairy related CPD amongst the participants, which ranged from 5 h to over 100 h undertaken in the last year. Our results also suggested that more experienced participants tended to suffer from being stuck in their comfort zone in a different way to less experienced participants. In particular, some senior partners/directors were no longer as proactive as they once were when it came to engaging with their clients over fertility. Reasons given for this included work load, management or other priorities, and a loss of intrinsic motivation. Therefore, finding ways for vets to flexibly incorporate high quality and engaging CPD into their workloads, would seem key.

The percentage breakdown of the clinical work within the practice will impact the exposure to dairy cattle fertility work. Reduced exposure will limit the vet's ability to gain knowledge and confidence. The likelihood that a practice would be willing to spend money on dairy fertility related CPD is also likely to be lower, as the cost benefit to the practice and the individual vet would not be as high compared to a practice with a higher percentage of dairy work. Suggestions for areas of specialisation within a practice have included lameness, young stock management, and fertility. These suggestions have been made for years and aim to maintain a high level of service to clients. Alongside this, it has been postulated whether there is a need to train omnicompetent vets at university (37). If this was not necessary, then it would increase species specific knowledge prior to graduation and provide higher levels of expertise through specialisation. However, in our view, mixed practices and mixed vets can continue to provide a high level of service to dairy clients, although mixed practices may face more challenges because time spent on the species and subject area will probably be less.

The relationship with the farmer was reported in this study to be crucial for vets to engage on the subject of fertility on the farm. The findings showed that it required good interpersonal and social skills to initially build that reputation. It has been shown previously that dairy farm vets often spend time on visits cultivating relationships with farmers and spend time discussing non-work related items to strengthen their bond (38). Gaining this relationship with the farmer allowed the vets to develop trust. Not having trust from the farmer was seen as a major barrier to being able to facilitate changes in practice. Participants perceived that the compatibility of personalities and good interpersonal skills lead to a good relationship and trust. This is supported with evidence from Svensson et al. (17) who showed that trust in the vet was key for farmers to adhere to advice given. However, greater emphasis was placed on the quality of the service provided from the vet to incur that trust rather than the relationship. Interestingly, trust as a construct has also been shown to be important for the uptake of services and adherence of treatment plans by the public in human health care settings (39).

Building trust with a farmer and understanding their priorities requires good communication skills and the ability to listen to and interpret the needs of the farmer. However, as we have previously highlighted, communication skills within the veterinary profession are sub-optimal at times and this may lead to poor engagement and adherence of advice. In addition, Bard et al. (40) proposes changing from a paternalistic to a mutualistic approach may aid and improve farmer engagement and behavioural change. This means that instead of the vet dictating a plan of action to a passive farmer, they are consulted and a mutually agreed plan is formulated and instigated.

Less experienced participants, in particular, perceived that they lacked the trust of their farmers. We hypothesise that this issue may have been exacerbated by the changing nature of the industry and the veterinary profession. In recent times, many smaller farm practices have amalgamated or have grown in size, employing a greater number of vets. This in turn may have resulted in a reduction in the number of visits a specific vet will have with a specific farm. This is likely to be experienced more often with less experienced vets, as many dairy farms will have a lead (senior) vet who usually carries out FHHPs and other preventative work, on their farm. It is therefore, arguably more important than ever, that less experienced vets are given as much opportunity to get on to farms regularly to build a strong relationship with the farmer. One way to help to achieve this is through a “buddy system” whereby assistants provide back up to lead (senior) vets on certain farms for fertility work. Furthermore, in this regard, we suggest mentorship support for vets in practice, at all stages of their career, may be useful, as well as formal leadership training for more experienced colleagues.

A concern for some participants was also the fear that they would be perceived to be “selling” to their farmers, such that the primary benefit for undertaking the advice would be for the vet and not the farmer. The concern for the participants was that this would erode the trust they had developed. This is echoed in a human healthcare setting, whereby financial incentives to doctors can be perceived negatively by the public and have been shown to lower their trust toward the profession (41). This concern by some of the participants over how a proportion of farmers may perceive their attempts to offer services and advice negatively, resulted in them restricting their engagement with all clients. This is an undesirable outcome for both clients and the vet. Identifying clients that are sceptical about a vet's underlying motivations, can aid in better targeted engagement such as economic modelling to highlight more robustly the cost-benefit to them of the services being offered.

It arose that many participants were highly motivated to engage with farmers that they had a good relationship with. However, they were far less likely to engage when the relationship was strained, if at all. Aphane et al. (42) showed that mutual respect was fundamental in effective team work within human health care. If that is not present, then there will always be a barrier to engagement. It seems reasonable to propose, therefore, that vets need to try to either overcome relationship barriers themselves, or facilitate colleagues into a position where they can effectively engage with the farmer. This is especially the case, given recent research in other clinical contexts has also highlighted the major importance of the vet-farmer relationship (43). Relationship barriers may arise for many reasons, including from a conflict of personalities or a previous engagement which has upset either the vet or the farmer. Prompt conflict resolution may improve trust and overall relationship with the farmer. Reflective relationships are stated as day 1 competences by the RCVS as of July 2020, thereby highlighting the importance of relationships for a vet in practice (44).

A strong theme throughout this study was the participants' job satisfaction when engaging successfully with farmers about fertility. This often arose from having a good working relationship which then turned into friendships, as well as from helping clients to improve their herd's fertility performance and subsequently enhancing cow health and welfare. Good relationships with clients has been recently reported as one of the main sources of job satisfaction for vets who stayed in their jobs (45). Participants also achieved significant job satisfaction by helping farmers to improve their fertility as they believed they were making their clients better off financially. This is in line with the work of Cake et al. (46) who showed that vets were predominantly motivated to help both animals and people.

In terms of the veterinary business, participants did not appear to be driven to engage with clients primarily for immediate financial gain for themselves, instead they perceived that if their clients were more profitable, then they would remain in farming for longer and be a source of longer-term income to the practice. And thus, be part of a more sustainable veterinary business model. In the UK, farm veterinary services have been put under greater pressure in recent times, in part due to a decrease in the number farms (47). Hence, ensuring that dairy farms are as efficient and profitable as possible will help to ensure future work and sustainability for the farm animal veterinary practice.

Not only did participants feel the threat of a shrinking market, they also felt the threat of vets in neighbouring practices. This belief has been found in other studies (9) which showed that the participants saw competition as a risk to their business. Within our study, the threat and competition from neighbouring practices tended to serve as a driver to increase practice standards and fuel further, proactive, engagement with clients. Research has shown that farmers prefer a proactive vet who can give good technical advice (16, 33). In addition, some participants were more concerned about competition arising from paraprofessionals and consultants, than they were from other vets. On this point, it is worth noting that all parties have the same aim, which is to aid the farmer. Therefore, working collaboratively with other professions, united by a shared goal, could prove productive for all stakeholders.

It is worth highlighting that fertility is a key determinant of the economics of a dairy business and determines whether every cow in the herd is culled, or not, every year. In our experience, and probably because of this, dairy farmers typically want to have good fertility and this is also likely to be the reason why, generally speaking, vets have traditionally had more involvement in this area in practice, compared to VHHP that is not specifically fertility related. Therefore, it could be argued that fertility is one of the easiest contexts in which to engage with dairy farmers. Yet interestingly, this study has shown that even in the context of fertility, there are numerous perceived and real barriers that exist and vets often find it challenging to engage with farmers to implement changes in practice. It raises the question of whether in non-fertility contexts, for which dairy farmers may be less intrinsically motivated, the challenges we have observed could be exacerbated.

Importantly, especially given our previous comments about the fundamental importance of fertility to dairy farmers, engagement, and undertaking fertility work on dairy farms were also seen by vets in this study as a gateway to discuss other herd health issues and disease problems on the farm. Furthermore, in general, participants felt that it was key for vets to change the business model of the practice, shifting the emphasis from emergency work to preventative medicine and advisory services. This is perhaps not surprising as a need to change farm veterinary business models has been proposed for a number of years (37). Not only will engagement on other health issues generate further revenue for the veterinary practice but it will enhance the reputation and trust between the two parties, further strengthening the vet-farmer relationship.

### Limitations

It is possible that there may have been some social desirability bias such that participants may have given some responses which reflected a desire to provide what they perceived to be the correct answer. Purposive sampling guaranteed a wide range of demographic characteristics to be included but may have introduced some bias. Due to the small sample size and localised geography of the interviews, this study should not be used to extrapolate and make generalisations about the veterinary profession as a whole.

## Summary

To our knowledge, this is the first study that has investigated vets' motivators and barriers for engaging with dairy farmers in order to help them to further their involvement in fertility management. The findings suggested four main themes that are involved in the vets' approaches to engaging with farmers. The theme “clinical baggage” arises from vets' previous negative experiences when attempting to engage with farmers. In some instances, this led to vets making assumptions about farmers' needs, wishes and behaviours, which affected their motivation to engage with their clients. Some vets also exhibited ageist stereotyping, believing that older farmers were less likely to implement their recommendations, which meant they were less likely to offer advice to older farmers. If advice is not acted upon by farmers, repeatedly offering it was problematic for some vets, causing fatigue and concerns about being perceived as a “salesperson.” Overall, in our view, some vets may become burdened, not helped, by their clinical experiences when it comes to facilitating change. Therefore, it is important for vets to reflect on how previous experiences of trying to facilitate change on farms may be influencing and perhaps weakening their current attempts to do so, and take steps to mitigate biases such as ageist stereotyping, as well as avoiding making assumptions about farmer intentions. The “stuck in the comfort zone” theme, highlighted that vets often accepted the current status quo of their work and become confined to their normal working pattern. Some vets lost enthusiasm over the course of their careers, while others lacked confidence to engage with farmers due to perceived gaps in their knowledge. Vets also reported farmers not perceiving their problems, and lack of farm data or facilities, as barriers to them having greater involvement with fertility. The “vet-farmer relationship” theme emphasised the importance of interpersonal skills and the need for vets to develop strong relationships and build trust with farmers. This was seen as critical for them to engage effectively and “go the extra mile” for their clients. The last theme, “money matters” explored vets' motivations to improve their clients' profitability and included the future sustainability of their own businesses.

### Implications for Research, Policy, and Practise

The key themes identified in this study can be used as a basis for further work which could include investigating the extent to which the motivators and barriers that have been identified here, occur on a wider scale within the profession. It is also essential that further training and support is provided for vets with regard to effectively engaging with farmers, at both undergraduate and postgraduate level. This study has helped to identify specific areas to target which includes mentoring networks for vets at all stages of their career, as well as enhanced training in communication, facilitation, and leadership skills. Identifying and overcoming conscious and unconscious bias toward factors such as age, personality and previous negative experiences is also key for vets at all stages of their career, along with clinical reflections specifically on farmer engagement, and we suggest that input from social scientists would be beneficial here. The findings from this research can also be used to help design and test future behaviour change interventions which would have the potential to help vets, farmers and their animals, as well as reducing the environmental impact of farming by improving the efficiency of production. Importantly, for vets working in clinical practice with high workloads, flexible opportunities and ring-fenced time to further undertake training and utilise support networks, is essential. It is notable that the support available for vets once they have qualified is very limited compared to their counterparts in other health professions such as medicine. Support in this area must be provided to vets working in clinical practice if improvements in production, animal health and welfare, and the environment are to be achieved.

## Data Availability Statement

The raw data supporting the conclusions of this article will be made available by the authors, without undue reservation.

## Ethics Statement

The studies involving human participants were reviewed and approved by University of Liverpool Veterinary Ethics Committee (reference number VREC791). The participants provided their written informed consent to participate in this study.

## Author Contributions

JB and HH together conceived the research idea and the study was designed by JB with input from EF, RS, and HH. JB recruited participants, conducted the interviews, and completed the initial coding of the transcripts. Higher-order analysis and theme development was led by JB with input from HH and EF. The manuscript went through several iterations, it was originally drafted by JB with comments from HH, EF, and RS. The final revision was made by HH. All authors read and agreed the final version.

## Funding

Our thanks go to the Barham Benevolent Foundation who very kindly funded the transcription costs for this research. The funder was not involved in any aspect of the study design, data collection, analysis, or reporting.

## Conflict of Interest

JB was employed by Craig Robinson Vets Ltd whilst simultaneously studying for a postgraduate qualification (Diploma in Bovine Reproduction at the University of Liverpool). The remaining authors declare that the research was conducted in the absence of any commercial or financial relationships that could be construed as a potential conflict of interest.

## Publisher's Note

All claims expressed in this article are solely those of the authors and do not necessarily represent those of their affiliated organizations, or those of the publisher, the editors and the reviewers. Any product that may be evaluated in this article, or claim that may be made by its manufacturer, is not guaranteed or endorsed by the publisher.
