# Supplementary material for: Motivations and Barriers for Veterinarians When Facilitating Fertility Management on UK Dairy Farms
Source: Front Vet Sci. 2021 Sep 29;8:709336. doi: 10.3389/fvets.2021.709336 (PMC8511516; doi:10.3389/fvets.2021.709336)
Supplement: Supplementary file 1 [file Data_Sheet_1.PDF]

Supplementary material: Interview Schedule

Section 1. Fertility work

Q. Please can you think of any dairy farmers in your practice for whom you, personally, are currently involved in doing their ‘routine fertility visits’. Please can you think specifically of one such client? Okay, thanks. Have you ever tried to engage with them to try to improve their fertility further? (e.g. by doing any investigations or challenging the status quo?

Prompt as required, for example:

- How did that go?
- Can you tell me what made you want to improve things?
- Why didn’t you try to engage with them?
- Why do you think one farmer followed your advice, and the other didn’t?
- Do you interact with different farmers differently to persuade them?

Ask about alternative responses if given.

Q. Okay thanks. Now, please can you now think of any dairy farmers in your practice who hardly ever, or never, ask for any help with their fertility. Have you personally ever tried to engage with any of these clients with a view to getting them to start having routine fertility visits? Okay, thanks. Please can you think specifically of one such client?

Prompt as required, for example:

- Can you tell me what made you want to do that?
- How did that go?
- In retrospect could you have done things differently?
- Why didn’t you try to engage with them?
- What about the situation would need to change in order for you to engage with them?

Ask about alternative responses if given.

Q. How have those experiences affected your views on offering your services to the rest of your clients? Are the experiences you have discussed generally true of your clients?

Q. How is your relationship with your farmers? How does this impact engagement with your clients about fertility?

Q. What is the value to the farmer of your input into the fertility of his cows?

Q. Does the practice market and publicise the fertility services on offer to your clients? How do they do that?

Q. Is there a difference of opinion on approach to fertility services within the practice i.e. treatment options for endometritis or data analysis? How does this impact you?

Q. What education have you received about fertility herd health management? How do you feel it has prepared you?

Q. What aspects of fertility work do you like or dislike?

Q. Is there any motivator or barrier to fertility work that you feel we haven’t covered that you would like to talk about?

Section 2. Demographics of participants and their veterinary practice.

1. Please could you tell me your age? (or just say, if you would prefer not to answer)

2. How many years have you been qualified as a veterinary surgeon?

3. How would you describe your role within the practice? (for example, new graduate, assistant, director, something else)

4. Is your practice a corporate or independent practice?

5. Do you have any post-graduate qualifications?

6. Is your practice a mixed practice, or ‘farm-only’ practice?

7. What percentage of your clinical time, do you spend working with dairy cattle?

8. How many regular fertility routines are you personally delivering?

9. How many vets in your practice work with farm animals?

10. How many dairy clients in total are served by your practice?

11. What is the range in size of the dairy herds served by your practice?

12. How many hours of dairy cattle related CPD, have you undertaken in the last year?
